# Forensic DNA Phenotyping: Genes and Genetic Variants for Eye Color Prediction

**DOI:** 10.3390/genes14081604

**Published:** 2023-08-10

**Authors:** Desiree Brancato, Elvira Coniglio, Francesca Bruno, Vincenzo Agostini, Salvatore Saccone, Concetta Federico

**Affiliations:** 1 Department Biological, Geological, Environmental Sciences, University of Catania, Via Androne 81, 95124 Catania, Italy; desiree.brancato@phd.unict.it (D.B.); elvira_con99@icloud.com (E.C.); francesca.bruno@unict.it (F.B.); federico@unict.it (C.F.); 2 Department Science and Technical Innovation, University of Eastern Piedmont, Viale Teresa Michel 11, 15121 Alessandria, Italy; vincenzo.agostini@uniupo.it

**Keywords:** eye color, externally visible characteristics, genetic polymorphisms, *OCA2* gene, *HERC2* gene, SNP rs12913832, melanogenesis, iris pigment

## Abstract

In recent decades, the use of genetic polymorphisms related to specific phenotypes, such as eye color, has greatly contributed to the development of the research field called forensic DNA phenotyping (FDP), enabling the investigators of crime cases to reduce the number of suspects, making their work faster and more precise. Eye color is a polygenic phenotype, and many genetic variants have been highlighted, with the major contributor being the *HERC2-OCA2* locus, where many single nucleotide variations (SNPs) were identified. Interestingly, the *HERC2-OCA2* locus, containing the intronic SNP rs12913832, the major eye color determinant, shows a high level of evolutionary conservation across many species of vertebrates. Currently, there are some genetic panels to predict eye color by genomic DNA analysis, even if the exact role of the SNP variants in the formation of eye color is still poorly understood, with a low level of predictivity in the so-called intermediate eye color. Many variants in *OCA2*, *HERC2*, and other genes lie in introns or correspond to synonymous variants, highlighting greater complexity in the mechanism of action of such genes than a simple missense variation. Here, we show the main genes involved in oculocutaneous pigmentation and their structural and functional features, as well as which genetic variants show the highest level of eye color predictivity in currently used FDP assays. Despite the great recent advances and impact of FDP in criminal cases, it is necessary to enhance scientific research to better understand the mechanism of action behind each genetic variant involved in eye color, with the goal of obtaining higher levels of prediction.

## 1. Introduction

Personal identification via a short tandem repeat (STR) profile has been considered the gold standard in forensic sciences for nearly 20 years. The discriminatory power of routine genetic assays based on STRs reached very high levels of reproducibility and safety of results. Even if the relevant technical advances in DNA profiling using STRs have reached very high levels, many important aims remain to be fulfilled to employ forensic investigations in humans by using a genetic approach [1,2].

Prediction of the phenotypic features of an individual by genotyping DNA polymorphisms opens a new era in forensic DNA phenotyping (FDP), which represents a great support to police investigations when matches in the DNA database or eyewitness descriptions are missing. FDP represents a historical and relevant improvement in forensic genetic technologies available to investigators in criminal cases. First, it shifts the focus of forensic science to the construction of evidence as a reliable and substantial contribution to investigations [3,4]. Second, FDP moves the focus from individualization of the suspects towards a group of ‘suspects’ sharing genetic origin or presenting specific externally visible characteristics (EVCs) [5,6]. Third, FDP uses single nucleotide polymorphisms (SNPs) that are associated with the development of certain visible traits. Thus, it is relevant to carry out genetic research on SNPs related to specific EVCs, which will also finalized in understanding their biological properties, namely the way they contribute to a specific phenotype by interfering with the expression of defined genes.

Currently, the use of genetic data in forensic applications to obtain a phenotypic appearance is not advanced enough [7]. Significant progress has been made with the implementation of next generation sequencing (NGS) technology to provide precious information about identity, biogeographic ancestry, paternity, and the possibility of adding massive parallel sequencing (MPS) with many DNA predictors, useful for forensic purposes such as FDP [8,9,10]. There are currently available a variety of genotyping assays based on MPS technologies developed to predict EVCs from DNA recovered in a crime scene, i.e., to reconstruct the appearance traits from highly decomposed remains, the biogeographic ancestry, the estimation of the age of the subject, and the age of bone remains, which certainly allow a more comprehensive approach toward the FDP applications [11,12,13]. The development of the MPS multiplex assay and the introduction of single-cell sequencing consent to enhance the EVC prediction, especially when low amounts of DNA or mixed traces were collected [14,15]. The FDP tool consists of two components: (1) a multiplex genotyping tool to analyze several DNA markers, and (2) a validated prediction model to obtain a high probability of appearance prediction. Moreover, the age estimation of the sample can also be obtained from epigenetic data by analysis of specific CpG sites [16,17], as can the identification of craniofacial features based on genetic variation [18]. However, there are some limitations to the recent and earlier studies on forensically DNA-based appearance, ancestry, and age prediction. First, the predictive power of DNA markers is still limited as the number of samples used has not yet reached sufficient levels. Second, the datasets used for building and validating the prediction model were also small in scale and dependent on the marker discovery data set [7].

Several genes are involved in eye color, and specific genetic polymorphisms have been identified by means of genome-wide association studies (GWAS), with the major contributor related to the *HERC2-OCA2* locus, where many SNPs were identified. The SNPs used for identification are actually in non-coding regions but function as FDP markers based on their association with protein-coding regions, highlighting greater complexity in the mechanism of action [19].

Forensic DNA phenotyping, including eye color prediction, is at its best, and the prediction model is being used as a routine tool by more forensic laboratories. However, we want to remind you of the complexity of DNA-based phenotype prediction due to the polygenic nature of human eye colors. Indeed, the accurate identification of a genotype from many polymorphic sites using, for example, NGS technologies to obtain a precise phenotype is only one step of the process, but the real current challenge lies behind data interpretation, joined to a molecular understanding of the action of each genetic polymorphism. To improve predictive performance, we show how different genes interfere with our eye color. As more extensively described by Maroñas et al. [20] and Dorgaleheh et al. [21], there are many genes involved in skin, hair, and eye color variation via melanosome biogenesis and the melanin biosynthetic pathway, with various effects on the pigmentation variation. Moreover, it should be stressed that a large number of SNPs related to eye color are located in introns, thus not directly determining a variation of the protein product of the gene involved.

These and other features should explain the variability in eye pigmentation within and between human populations, and it is therefore relevant to understand the molecular details associated with individual SNPs. In this regard, melanocyte cell lines could be extensively used as in vitro models to investigate the mechanism of action related to the polymorphisms in the genes we show in the present review. Most of the genes and their polymorphisms are expressed in the melanocytes, and a number of them are involved, directly or indirectly, in the melanogenesis pathway. Therefore, in order to establish a direct genotype/phenotype correlation, not only population studies are necessary to identify specific genotypes in some populations, but also advances in in vitro studies on human melanocyte cells could represent a useful model for the study of the polymorphisms most involved in the determination of eye color. An in vitro study model based on human melanocytes, using various molecular approaches to investigate each locus and the related SNPs, will become the crucial next step in understanding the genetic basis of eye color for forensic purposes.

## 2. The Genetic Basis of Eye Color: *OCA2* and *HERC2* Polymorphisms

**Figure 1:**
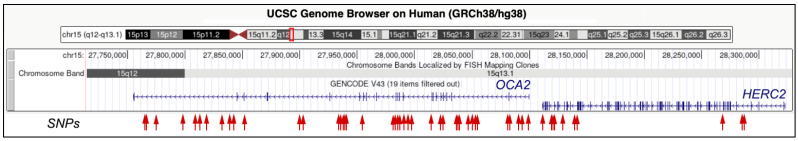
Genomic organization of the *OCA2* and *HERC2* genes. *OCA2* and *HERC2* are very close to each other on human chromosome 15, with the 3′-end of *HERC2* adjacent to the promoter region of the *OCA2* gene. The red box in the chromosome ideogram indicates the position of the *OCA2*-*HERC2* locus. Image from the UCSC Genome Browser (http://genome.ucsc.edu, accessed on 2 June 2023). At the bottom, the positions of the SNPs related to eye color, with details described in this section, are indicated by red arrows.

The eye color shows variable pigmentation depending on differences in the amount, type, and distribution of melanin obtained by the melanogenesis process in melanocytes [22]. To understand the range of phenotypic variation in pigmentary traits, the regulation of melanogenesis must be considered, as its multi-step process is under genetic control involving multiple genes, which overall affect the outcome of eye color in humans. In the past, *OCA2* was considered the main gene involved in eye color [23]. However, it was shown that the *HERC2* gene, positioned very close to *OCA2* (Figure 1), plays a pivotal role in iris color determination, influencing the expression of *OCA2* [24,25].

Gene interactions are often involved in the determination of complex traits; thus, the relevance of a single gene on complex phenotypic features cannot be determined in a simple way due to epistatic effects among different genes [26], even if the interaction between *HERC2* and *OCA2* genes cannot be compared to a classic epistatic effect. Indeed, *HERC2* influences the *OCA2* gene at the DNA level by modulating its transcriptional activity [27], differently from other genes found to interplay in the determination of eye color in a different way [25]. Indeed, in addition to the major eye color determinants *HERC2*/*OCA2*, with more than 50 SNPs related to eye color, other genes were shown to be involved in eye color (Table 1). Together with *HERC2* and *OCA2*, these genes are the best predictors for blue, brown, and intermediate eye color, bearing in mind that eye color phenotyping has followed several proposals, including light vs. dark and blue vs. brown [23,28,29]. Only recently was another categorization proposed: blue (comprising light to dark blue), intermediate (comprising green and hazel), and brown (comprising light to dark brown).

### 2.1. OCA2 Gene

The *OCA2* gene (NM_000275.3) is localized at the chromosomal band 15q13.1 and is composed of 24 exons spanning a region of 345 kb. It codes a protein of 838-aa (aa: aminoacids) (NP_000266.2), namely the human P protein, which is a homologue to the mouse pink-eyed dilution gene (p), an integral melanosomal membrane protein that contains 12 transmembrane-spanning regions and may stabilize the traffic of melanosomal proteins such as tyrosinase, regulate melanosomal pH, or serve as a melanosomal tyrosinase transporter. Altered P protein may therefore affect pigmentation characteristics via altered tyrosinase or tyrosinase bioavailability and function [30].

This function appears to be conserved in an evolutionary manner, as mutations of *OCA2* result in oculocutaneous albinism, an autosomal recessive disorder that is characterized by reduced or absent biosynthesis of melanin pigment in melanocytes of the skin, hair follicles, and eye [31]. The genetic variability of *OCA2* appears to be largely associated with eye color, with a contribution of 74% to the total phenotypic variance of eye color. Thus, the *OCA2* locus is considered the major genetic determinant for blue-brown eye color, and currently many polymorphisms of this gene have been largely studied and identified as directly responsible for variation in iris pigmentation [32]. 44 apparently nonpathogenic variant alleles of the *OCA2* gene have been identified with distinct frequencies in varied populations, which explains differences in pigmentation phenotypes among ethnic groups [33]. The large number of these SNPs (37 out of 44) are in the intronic regions of *OCA2*, and only six are in the coding regions, with one of these being a synonymous substitution (Table 2).

**Table 2:** Genetic properties of the main SNPs related to eye color.

Gene	SNP-ID ^(a)^	Allele	Variant	Chr.	Pos. (nt)	Variant Type
*ASIP*	rs1015362	C	T	20q11.22	34,150,806	5′UTR
*ASIP*	rs6119471 *	C	G	20q11.22	34,197,406	Intron
*ASIP*	rs6058017	A	G	20q11.22	34,269,192	3′UTR
*HERC2*	rs1129038 *	C	T	15q13.1	28,111,713	Intron
*HERC2*	rs191109490	A	C	15q13.1	28,120,403	Intron
*HERC2*	rs12913832 *	A	G	15q13.1	28,120,472	Intron
*HERC2*	rs7183877 *	C	A	15q13.1	28,120,587	Intron
*HERC2*	rs3935591	T	C	15q13.1	28,128,866	Intron
*HERC2*	rs551217952	A	C	15q13.1	28,139,387	Intron
*HERC2*	rs11636232 *	C	T	15q13.1	28,141,480	Syn.: Gln3989Gln
*HERC2*	rs916977	T	C	15q13.1	28,268,218	Intron
*HERC2*	rs1667394 *	C	T	15q13.1	28,285,036	Intron
*HERC2*	rs12592730	G	A	15q13.1	28,285,213	Intron
*IRF4*	rs12203592 *	C	T	6p25.3	396,321	Intron
*MC1R*	rs2228479	G	A	16q24.3	89,919,532	Missense: Val92Met
*MC1R*	rs1805007	C	T	16q24.3	89,919,709	Missense:Arg151Cys
*MC1R*	rs1805008	C	T	16q24.3	89,919,736	Missense: Arg160Trp
*MC1R*	rs885479	G	A	16q24.3	89,919,746	Missense: Arg163Gln
*MC1R*	rs1805009	A	C	16q24.3	89,920,138	Missense:Asp294His
*OCA2*	rs1498519	G	T	15q13.1	27,766,505	Intron
*OCA2*	rs4778177	C	T	15q12	27,766,748	Intron
*OCA2*	rs8023340	A	G	15q12	27,775,242	Intron
*OCA2*	rs1874835	G	T	15q12	27,799,154	Intron
*OCA2*	rs924314	G	A	15q13.1	27,809,200	Intron
*OCA2*	rs924312	C	G	15q13.1	27,812,717	Intron
*OCA2*	rs74409036	G	A	15q13.1	27,818,606	Intron
*OCA2*	rs4778190	T	C	15q13.1	27,832,014	Intron
*OCA2*	rs2036213	C	A	15q13.1	27,839,034	Intron
*OCA2*	rs78544415	C	T	15q13.1	27,842,634	Intron
*OCA2*	rs1004611	G	A	15q13.1	27,852,132	Intron
*OCA2*	rs2871886	T	C	15q13.1	27,899,878	Intron
*OCA2*	rs3099645	G	T	15q13.1	27,902,213	Intron
*OCA2*	rs977588	C	A	15q13.1	27,934,160	Intron
*OCA2*	rs977589	C	T	15q13.1	27,934,457	Intron
*OCA2*	rs1375170	G	T	15q13.1	27,939,008	Intron
*OCA2*	rs1448490	C	A	15q13.1	27,939,381	Intron
*OCA2*	rs2594935	G	A	15q13.1	27,939,892	Intron
*OCA2*	rs1800414	T	C	15q13.1	27,951,891	Missense: Hys615Arg
*OCA2*	rs728405	C	A	15q13.1	27,954,707	Intron
*OCA2*	rs74653330	C	T	15q13.1	27,983,407	Missense: Ala481Thr
*OCA2*	rs1900758	C	T	15q13.1	27,984,951	Intron
*OCA2*	rs121918166	C	T	15q13.1	27,985,101	Missense: Val443Ile
*OCA2*	rs1800407 *	C	T	15q13.1	27,985,172	Missense: Arg419Gln
*OCA2*	rs10852218	T	C	15q13.1	27,986,647	Intron
*OCA2*	rs1800404	C	T	15q13.1	27,990,627	Syn: Ala355Ala
*OCA2*	rs735066	A	G	15q13.1	27,993,749	Intron
*OCA2*	rs1800401	G	A	15q13.1	28,014,907	Missense: Arg305Trp
*OCA2*	rs2305252	C	T	15q13.1	28,022,192	Intron
*OCA2*	rs3794606	A	C	15q13.1	28,023,862	Intron
*OCA2*	rs4778232 *	C	T	15q13.1	28,036,619	Intron
*OCA2*	rs1448485	G	T	15q13.1	28,037,595	Intron
*OCA2*	rs8024968 *	C	T	15q13.1	28,038,543	Intron
*OCA2*	rs1375164	C	T	15q13.1	28,046,666	Intron
*OCA2*	rs1597196	G	T	15q13.1	28,049,776	Intron
*OCA2*	rs895828	G	C	15q13.1	28,052,887	Intron
*OCA2*	rs895829	T	C	15q13.1	28,052,911	Intron
*OCA2*	rs4778137	C	G	15q13.1	28,082,689	Intron
*OCA2*	rs72714116	C	T	15q13.1	28,083,061	Intron
*OCA2*	rs4778138	A	G	15q13.1	28,090,674	Intron
*OCA2*	rs4778241 *	A	C	15q13.1	28,093,567	Intron
*OCA2*	rs7495174	T	G	15q13.1	28,099,092	Intron
*SLC24A4*	rs12896399 *	G	T	14q32.12	92,307,319	5′UTR
*SLC24A5*	rs1426654	A	G	15q21.1	48,134,287	Missense: Thr111Ala
*SLC45A2*	rs16891982 *	C	G	5p13.2	33,951,588	Missense: Leu374Phe
*SLC45A2*	rs26722	C	T	5p13.2	33,963,765	Missense: Glu272Lys
*TYR*	rs1393350 *	G	A	11q14.3	89,277,878	Intron
*TYR*	rs1126809	G	A	11q14.3	89,284,793	Missense: Arg402Gln
*TYRP1*	rs1325127	C	T	9p23	12,668,328	5′UTR
*TYRP1*	rs1408799	T	C	9p23	12,672,097	5′UTR
*TYRP1*	rs62538956	T	C	9p23	12,679,244	5′UTR
*TYRP1*	rs35866166	T	C	9p23	12,698,471	Syn: Ser243Ser
*TYRP1*	rs2733832	C	T	9p23	12,704,725	Intron
*TYRP1*	rs683	C	A	9p23	12,709,305	3′UTR

^(a)^ Data from NCBI dbSNP (www.ncbi.nlm.nih.gov/snp/, accessed on 2 June 2023). * SNPs currently used in forensic DNA prediction systems (see Section 4 for details, Table 3).

**Table 3:** SNPs used in forensic DNA prediction systems.

Gene	SNP-ID	Walsh et al. [34] ^(a)^	Allwood et al. [35]	Ruiz et al. [36]	Hart et al. [37]
*ASIP*	rs6119471				X
*HERC2*	rs1129038		X	X	
*HERC2*	rs12913832	X		X	X
*HERC2*	rs7183877			X	
*HERC2*	rs11636232			X	
*HERC2*	rs1667394			X	
*IRF4*	rs12203592	X		X	X
*OCA2*	rs1800407	X	X	X	
*OCA2*	rs4778232			X	
*OCA2*	rs8024968			X	
*OCA2*	rs4778241			X	
*SLC24A4*	rs12896399	X	X	X	X
*SLC45A2*	rs16891982	X		X	X
*TYR*	rs1393350	X	X	X	

^(a)^ IrisPlex system. X indicate which SNPs are used in the different prediction systems. The genomic features of the above SNPs are shown in Table 2.

Three SNPs, rs7495174:A/G, rs6497268 (merged into rs4778241):A/C, and rs11855019 (merged into rs4778138):A/G in intron 1 underlies the genetic linkage of blue/brown eyes, while two *OCA2* coding-region variant alleles—rs1800401:G/A->Arg305Trp and rs1800407:C/T->Arg419Gln—were shown to be associated with brown and green/hazel eye colors, respectively [11]. It was hypothesized that an epistatic effect of rs1800407:C/T and rs12913832:A/G would decrease pigmentation level and increase the prediction accuracy of intermediate eye color by also considering another *HERC2* SNP, rs1129038:C/T, which is found to be in linkage with rs12913832:A/G [36,38,39].

### 2.2. HERC2 Gene

The *HERC2* (NM_004667) gene is located on chromosomal band 15q13.1, adjacent to *OCA2*, and contains 98 exons spanning a region of 211 kb. It is a member of the *HERC* protein family, endowed by the presence of at least one regulator of chromosome condensation 1 (RCC1)-like domain (RLD) and a homologous E6AP carboxy terminus (HECT) domain characteristic of a group of ubiquitin ligases. Mutations on the *HERC2* gene have been described as being involved in severe neurological conditions, including syndromes of intellectual disability, autism, and a variable neurological deficit named Angelman syndrome [40]. *HERC2* is overexpressed in many tissues, and genetic variations in this gene are associated with pigmentation variability, but cellular activity and its regulation remain poorly understood.

In 2008, three studies highlighted the role of *HERC2* and described the SNP located in intron 86, rs12913832:A/G, as a major eye color predictor strongly associated with *OCA2* expression levels [13,24,25,41]. More recently, it was experimentally demonstrated that this SNP acts as an enhancer, regulating *OCA2* transcription by distantly modulating chromatin folding [27].

### 2.3. Model for OCA2 Gene Regulation

**Figure 2:**
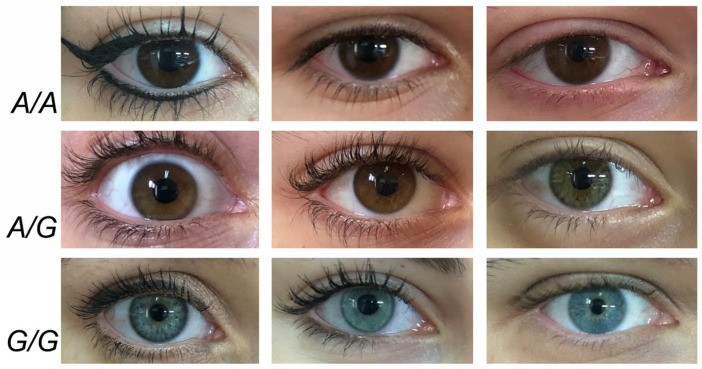
Phenotypic effect of the SNP rs12913832:A/G in eye color. Representative eye colors related to the three genotypes of SNP rs12913832. A/A homozygous genotype is related to the darker brown color (upper images). On the contrary, the G/G homozygous genotype is related to the lighter blue eye color (bottom images). The heterozygous A/G genotype is related to intermediate color of the eyes, namely by the iris with a lesser amount of melanin (respecting the A/A genotype) determining a lesser intensity of the brown or green-hazel color. These are unpublished images by the authors.

Different reports recognized that the SNP rs12913832:A/G accounted for blue-brown eye color (Figure 2).

Additional evidence that supports the pivotal role of intron variants related to the pigmentary trait is the high level of evolutionary conservation of rs12913832 across many species of vertebrates. In fact, the rs12913832 polymorphism is contained in a region with 77% sequence identity between humans and mice, and specifically, it is located in a conserved 11-base sequence GACA(T/C)TTAAT, suggesting that this region may represent a consensus binding site for the helicase-like transcription factor (HLTF) [25]. HLTF is a member of the SWI/SNF family and is implicated in many processes involving chromatin remodeling to permit the correct access of the transcriptional machinery. Thus, it was proposed a molecular basis for eye color determination involving the rs12913832:A allele via chromatin remodeling that leads to OCA2 expression and acts in the maturation pathway of the melanosome producing brown iris [25]. The rs12913832:G allele, on the other hand, is related to a closed chromatin structure and is then unavailable for transcription of the OCA2 locus. This OCA2 transcriptional repression in human melanocyte cells results in the appearance of a light blue iris.

Experimental evidence to support the regulatory role of *HERC2* has been provided by demonstrating that rs12913832 functions as a human melanocyte-specific enhancer element that regulates *OCA2* transcription using various molecular approaches [27]. In particular, rs12913832 acts as an enhancer, communicating with the *OCA2* promoter via a long-range chromatin loop, and this activity is mediated by the transcription factors HLTF, LEF1, and MITF.

## 3. Other Genes Related to the Eye Color

### 3.1. TYR Gene

**Figure 3:**
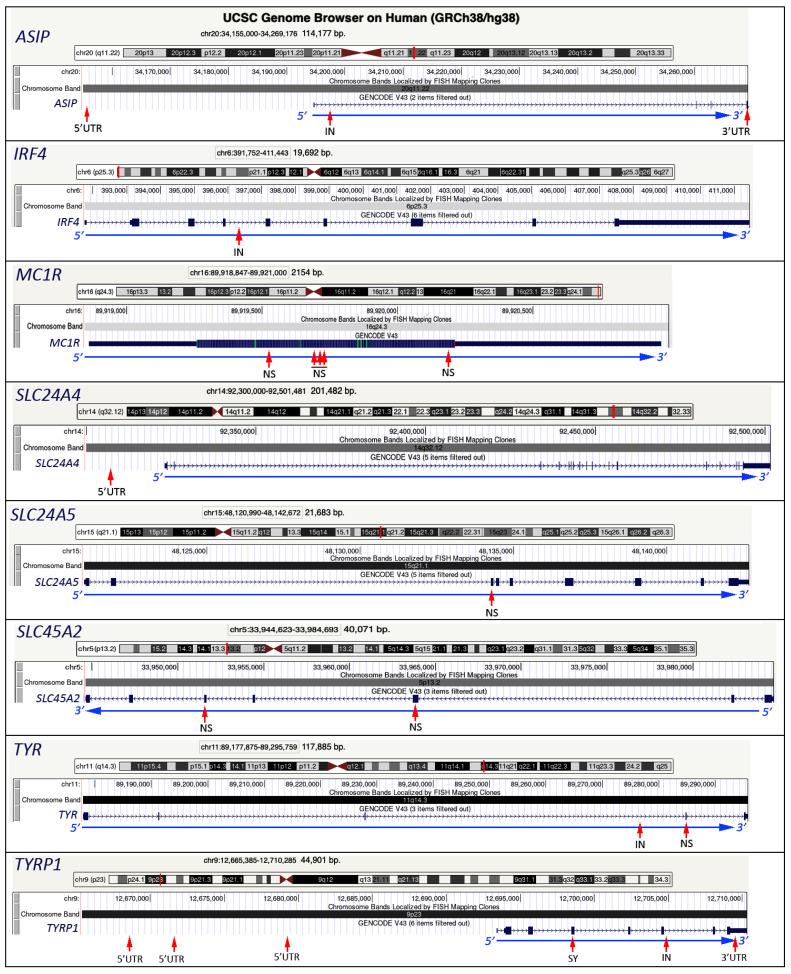
Genomic organization of the main genes involved in eye color other than *HERC2*/*OCA2*. Images from the UCSC Genome Browser (http://genome.ucsc.edu, accessed on 26 July 2023), with the transcriptional direction indicated at the bottom of each gene. The position and the features of the SNPs related to the eye color (see Table 2) are indicated by the red arrows. IN: intron; SY: synonymous substitution; NS: non-synonymous substitution; 5′UTR and 3′UTR: untranslated regions at 5′ and 3′, respectively.

*TYR* (Tyrosinase) (NM_000372) was one of the first human pigmentation genes identified and characterized because mutations in this gene are related to OCA1 albinism, a group of autosomal recessive disorders characterized by reduced production of melanin. *TYR* is located on chromosome 11q14.3 and consists of 5 exons of 117 kb (Figure 3), coding for a copper-binding protein with 529 amino acid residues, a melanosomal membrane-bound glycoprotein with a native molecular weight of 55 kD [42,43]. Tyrosinase plays an important role in melanin biosynthesis, catalyzing the limiting steps that convert L-tyrosine to L-DOPA and then to DOPAquinone. Polymorphisms within the *TYR* locus, i.e., rs1126809:G/A in exon 4 determining the Arg402Gln variant, occur at high frequency but are not immediately correlated with pigmentation phenotypes. In fact, the relationship of these SNPs to normal variation in pigmentary variation in Europeans has been studied by numerous research groups, with reported associations for lighter eye color and brown hair color, but such effects are not always detectable [44]. Another SNP, rs1393350:G/A, strongly correlated with the above SNP, rs1126809, is correlated with skin, hair, and eye color. The rs1393350:A polymorphism is associated with blue versus green eye color, blond versus brown hair, and skin sensitivity to the sun. However, despite the pleiotropic effect of rs1393350 on pigmentation traits, no positive selection based on population divergence or extended haplotype homozygosity was found [26].

### 3.2. TYRP1 Gene

Another gene, located on the 9p23 chromosomal band, involved in pigmentation differences is *TYRP1* (Tyrosinase-related protein 1) (NM_000550), which consists of eight exons spanning 17 Kb (Figure 3). The protein encoded by *TYRP1* was described for the first time in 2003 as a tyrosinase-like enzyme that takes part in melanin biosynthesis [45]. Mutations in the *TYRP1* gene cause oculocutaneous albinism (OCA3). Normally, TYRP1 is involved in the pathway of eumelanin production, and two *TYRP1* variants, rs1408799:T/C and rs683:C/A, are relevant in eye color prediction [29]. The rs1408799 is not in the regulatory region, but it shows an association with the *HERC2* SNP rs12913832:GG genotype. Also, two other variants, rs62538956:T/C, located in a melanocyte-specific enhancer, and rs35866166:T/C, located in a melanocyte-specific promoter region, are involved in eumelanin production. In general, a downregulation of *TYRP1* expression results in less pigmentation and blue eyes [46].

### 3.3. SLC45A2 Gene

In recent years, *SLC45A2* (solute carrier family 45 member 2) (NM_016180), also called *MATP* (membrane associated transporter protein), was added to the group of genes (comprising *MC1R*, *OCA2*, and *ASIP*) related to pigmentation. Mutations in this gene, which encodes the membrane-associated transporter protein, have recently been associated with OCA4 albinism [33].

Originally described as an antigen in human melanoma (AIM1), the 530 amino acid MATP protein was detected in melanocyte cells of several normal tissues [47]. Subsequently, it was renamed *SLC45A2*, a gene with a genomic size of about 40 kb, composed of 7 exons, and located on chromosomal band 5p13.2 [48] (Figure 3). The MATP protein shares 82% identity with the orthologous mouse protein, has 12 transmembrane domains, and is transcriptionally regulated by MITF by an indirect mechanism [49,50].

Two nonsynonymous polymorphisms, rs26722 (c.814C/T p.Glu272Lys) and rs16891982 (c.1122C/G p.Leu374Phe), are relevant candidates as susceptibility variants because they show distinct population frequency distributions and have been strongly associated with dark eye pigmentation. Both polymorphisms result in aminoacid substitutions potentially altering the function of MATP, which may in turn affect cytoplasmic interactions with other proteins involved in pigmentation [48]. An increasing of rs16891982:G frequency was seen as eye pigmentation got progressively darker and evolutionary conservation of the G-allele across different species. This allele is considered the ancestral one, being observed at high frequency in West Africans [51]. The G-allele favors optimal eumelanin production; indeed, the leucine amino acid within the transmembrane region of the protein, having a relevant role in proton transport, creates an intra-melanosomal pH for optimal tyrosinase activity [49].

The C-allele determines a decrease in functionality of the protein that alters the intracellular trafficking of melanosomal proteins and decreases melanin production [48]. This functional difference seems to be correlated with a better absorption of ultraviolet β rays (UVBs) in individuals living at high latitudes, which is useful for the synthesis of vitamin D3. On the contrary, in equatorial areas, this allele seems to enhance the harmful effects of UVR, thus eliminating any possible beneficial effect [52].

### 3.4. SLC24A4 Gene

*SLC24A4* (solute carrier family 24 member 4) (NM_153646) is located in 14q32.12 is a gene that belongs to the same family as *SLC24A5* (see below), contains 17 exons covering 178 kb (Figure 3), and encodes a member of the potassium-dependent sodium/calcium exchanger protein family. Genetic variants in *SLC24A4* contribute to variation in the skin, hair, and eye pigmentation. In particular, rs12896399:G/T was initially associated with eye color, and the greatest difference in allele frequency for rs12896399 was between blue-eyed and green-eyed individuals [26]. The masking effect of *HERC2* SNP rs12913832 on rs12896399 in determining blue eye color was revealed [53]. It was shown that the GG, GT, and TT genotypes in rs12896399 are strictly related to rs12913832. Indeed, among these genotypes, rs12896399:TT was classified with ‘high-probability’ of having blue eyes when associated with the GG genotype in rs12913832. The allele A in rs12913832 in *HERC2* masks this effect, and, independently of the genotype in rs12896399, individuals are classified into the group of ‘low-probability’, to have blue eye color [26].

### 3.5. SLC24A5 Gene

The *SLC24A5* (solute carrier family 24 member 5) (NM_205850) gene is located on chromosomal band 15q21.1 with 9 exons covering 22 kb (Figure 3) and encodes the protein NCKX5 that functions as an ion carrier, involved in the control of pH within the melanosomes [54]. Non-synonymous single nucleotide polymorphisms in these genes have been associated with pigmentation color variation in homogeneous populations [55]. The rs1426654 is characterized by a substitution of an adenine to a guanine in the 3rd exon of the gene (A/G), resulting in the substitution of threonine by alanine at position 111 of the protein (Thr111Ala); this reduces efficiency in the transport of ions and in the production of pheomelanin [56]. The polymorphic homozygous genotype of rs1426654:A showed the strongest association with blue eyes and varies in frequency between populations. It was postulated that several European populations carry the polymorphic allele A of *SLC24A5* (Thr111) in high frequency (98–100%), while the ancestral allele G (111Ala) is markedly present in African populations (93–100%) [54]. In 2014, the SNP rs1426654 was described as the first marker to distinguish black from non-black traits, thus highlighting the importance of SLC24A5 in forensic applications [57]. Moreover, it shows a population-specific pattern like p.Leu374Phe of *SLC45A2*, with the derived A allele fixed in Europeans [55].

### 3.6. ASIP Gene

Given the role *ASIP* (NM_001672) plays in the switch from eumelanin to phaeomelanin production, it is one of many strong candidates to explain inter-individual variation in human pigmentation. *ASIP* is located on 20q11.22, with 4 exons that cover a region of 28 kb (Figure 3), encodes the Agouti signaling protein, a 132-amino-acid protein, and is expressed in several tissues. It plays a role in the regulation of pigmentation by inhibiting eumelanin synthesis and inducing the production of pheomelanin [58,59]. In *ASIP*, the SNP rs6058017 is in the 3′ UTR of the gene, 25 bp downstream of the codon STOP, and corresponds to a substitution of the adenine to a guanine (A/G). The proposed mechanism of action for the *ASIP* rs6058017:G SNP involves reduced mRNA stability and premature degradation of the transcript, exerting less antagonism toward *MC1R*, which will bind to the α-Melanocyte-stimulating hormone (α-MSH), leading to the production of eumelanin, namely the dark eyes [60]. The allele A of the above polymorphism is present at high frequencies (>75%) in the European population, and the allele G (the ancestral one) is found in East Asian (over 60%) and African–American (40%) populations [61].

### 3.7. MC1R Gene

The *MC1R* (melanocortin-1 receptor) gene (NM_002386) is intronless and spans about 2 kb on the 16q24.3 chromosomal band (Figure 3), coding for a melanocyte-stimulating hormone receptor (MSHR), a G protein-coupled transmembrane receptor that is highly polymorphic and responsible for intraspecific and interspecific color variation [62,63]. Several features make it ideal for population genetic analysis. First, because it is a small gene, it is well adapted to complete sequence analysis. Second, the functional role of MC1R within biochemical melanogenesis pathways is well characterized. Third, several studies provided considerable details about genotype-phenotype associations of *MC1R* variants, three of which were associated with red hair and increased sensitivity to UV radiation [64]. These three variants (Arg151Cys, Arg160Trp, and Asp294His) all bind melanocyte-stimulating hormone but show lower intracellular ability to activate adenylate cyclase, leading to a switch in production from eumelanin to pheomelanin [65,66]. A total of 38 SNPs spanning a 1-Mb region of strong linkage disequilibrium (LD) showed an association with red hair, skin sensitivity to the sun, and freckles, reaching genome-wide significance. More than 30 non-synonymous mutations have been described in populations of European ancestry that affect the normal function of the *MC1R* gene product [67]. Two non-synonymous *MC1R* mutations, namely rs1805007:C/T and rs1805008:C/T, are common in European populations and have a major effect on normal differences in pigmentation. Their mutated T alleles were correlated with the allele rs1805009:A. Although this gene shows a strong correlation with light skin color, freckles, and red hair, no studies have shown at the molecular level the possible effect of *MC1R* on eye color variation.

### 3.8. IRF4 Gene

*IRF4* (6p25.3) (NM_002460), belonging to the interferon (IFN) regulatory factors, was first linked to pigment cells and then with human pigmentation and covers a region of 20 kb with 9 exons in the chromosomal band 6p25.3 [26,68,69] (Figure 3). In 2013, it was demonstrated that the melanocyte regulator MITF activates expression of the *IRF4* gene depending on the presence of the transcription factor activator protein α (TFAP2A). A sequence variant overlaps the TFAP2A binding site, affecting binding by this transcription factor and consequently lowering *IRF4* expression [70]. Since the MITF and IRF4 proteins cooperatively activate expression of Tyrosinase, a direct link between a polymorphism in the intronic region of the *IRF4* gene and reduced expression of an enzyme essential for pigmentation has been established. In particular, the rs12203592:C/T located in intron 4 acts as a melanocyte-specific enhancer element, and the rs12203592:T allele reduces its activity [70]. Two years later, in 2015, another group proved that the rs12203592 enhancer interacts with the *IRF4* promoter through an allele-dependent chromatin loop, and allele-specific activation of *IRF4* transcription is stabilized by a loop from the rs12203592 enhancer to another regulatory element in *IRF4* [71]. Initially, the rs12203592 SNP within 69.7 kb of two additional SNPs (rs4959270 and rs1540771) was identified as a determinant of hair color in women of European ancestry by a GWAS [26]. Subsequently, the SNP was also associated with lighter skin color, less tanning ability, and blue/light eye color [69]. Although it is included in the IrisPlex assay (see below), no association was found between rs12203592 and eye color in individuals with the rs12913832:GG genotype because these variants were associated only categorically but not quantitatively with eye color [46].

## 4. Gene Panels to Predict Eye Color

Over the years, progress has been made to establish eye color prediction. The first two studies used 33 SNPs from the *OCA2* gene to predict eye color, correctly classifying 8% of eye color among 1000 samples [28]. Nine SNPs from six genomic regions (*OCA2–HERC2*, *TYR*, *SLC24A4*, *KITLG*, *IRF4*, and *MC1R*) were significantly associated with eye color by the first GWAS on several Europeans [26]. Based on the DNA predictions, individuals with a low probability of having brown eyes showed blue eyes, while individuals with a high probability of having brown eyes were dark-eyed.

In 2008, a specific haplotype based on 13 SNPs from the *OCA2–HERC2* locus, including *HERC2* rs12913832 and rs916977, was identified in individuals with blue eyes [24,25,41]. *HERC2* rs916977 was highlighted as a major eye color predictor by a microarray panel of GWAS, and formal DNA-based prediction of eye color was performed using only 3 SNPs, including *HERC2* rs916977, *OCA2* rs11855019, and *OCA2* rs7495174 [24]. The authors obtained prediction accuracies expressed as AUC (area under the receiver characteristic operating curve) of approximately 0.8 for brown and blue eye colors, respectively (where 0.5 means random prediction and 1.0 means completely accurate prediction); most of the eye color predictive value was provided by the *HERC2* rs916977 [29].

In 2009, another DNA-based prediction study was published using 37 SNPs from eight different pigmentation genes with the aim of investigating their eye color predictive capacity in Dutch Europeans [29]. A single SNP, *HERC2* rs12913832, achieving AUC values of 0.899 for brown and 0.877 for blue, expressed the highest predictive value. They also proposed a minimal set of eye color DNA predictors made up of six SNPs from six pigmentation genes (*HERC2:* rs12913832, *OCA2:* rs1800407, *SLC24A4:* rs12896399, *SLC45A2:* rs16891982, *TYR:* rs1393350, and *IRF4:* rs12203592), with AUC values of 0.93, 0.91, and 0.72 for brown, blue, and intermediate eye color, respectively, in Dutch Europeans used for model validation.

Another study tested the predictive effect on pigmentation variation of 75 SNPs from 24 genes on a mixed population of Europeans and non-Europeans [72]. Only three SNPs from three pigmentation genes, rs12913832 (*HERC2*), rs16891982 (*SLC45A2*), and rs1426654 (*SLC24A5*), provided an accurate prediction calculated by multiple linear regression models, with the highest value reached by *HERC2* rs12913832 alone. However, the prediction outcomes are difficult to interpret as creating SNPs using a mixed population represents a challenge in separating ancestry effects from eye color.

Mengel-From et al. [73] also confirmed the predictive value of *HERC2* rs12913832 and *OCA2* rs1800407 for eye color in Danish Europeans, which together with SNPs rs1129038 and rs11636232 (in the *HERC2* gene) are in strong linkage disequilibrium in Europeans (rs12913832 and rs1800407 overlap with the best 6 SNPs from [29]).

In the above preliminary studies, to characterize the predictive value of the SNPs, not all used the same genes or the same SNPs. For example, Frudakis et al. [28] analyze only SNPs in *OCA2* that partially overlap with those studied by Sulem et al. [26] and by Valenzuela et al. [72]. However, Valenzuela et al. [72] include analysis of polymorphisms in different genes not mentioned by any other authors, i.e., *GPR143*, *HPS*, and *LYST*. In general, the overlap is more relevant for SNPs in the *OCA2-HERC2* locus, together with other SNPs present in genes included in the current main prediction systems shown in Table 3.

Based on previous discoveries and the observed SNP prediction values, the IrisPlex system was developed, representing the first forensically validated phenotyping system [34]. It includes a multiplex genotyping assay of the six eye color-predicting SNPs from *HERC2*, *OCA2*, *SLC24A4*, *SLC45A2*, *TYR*, and *IRF4* genes (Table 3) and implements the prediction model from [29], which is accessible as an Excel sheet that provides the probability of eye color from the genotypes of the SNPs used in this system. It provided a high level of discrimination between blue and brown eye colors (>90%). The IrisPlex system was tested to predict eye color in 940 worldwide DNA samples; different eye colors were only predicted in European samples, while only brown eyes were predicted in samples from regions such as East Asia, Africa, Oceania, and Native Americans in accordance with the known global distribution of eye color categories [74].

A subsequent validation of the model on Europeans from seven countries was performed to prove the robustness of the IrisPlex model [75]. Currently, the IrisPlex assay was tested by the International Society for Forensic Genetics (ISFG) in a multi-center training involving several laboratories with different levels of specific experience and was found to be easy to implement and highly trusted [76].

At present, all 6 IrisPlex SNPs are included in a commercial tool allowing to deduce biogeographic ancestry, appearance, relatedness, and sex from genome-wide SNPs [77]. Subsequent to the IrisPlex’s publication, other SNP sets with partially overlapping markers have been proposed for eye color prediction. In fact, in 2013, a group of researchers tested other SNPs from 10 pigmentation genes in a sample of European and non-European origin and proposed a set of 4 SNPs in the *SLC24A4*, *OCA2*, *TYR*, and *HERC2* genes (Table 3). The first three genes overlap with IrisPlex, while the latter is in strong linkage disequilibrium (LD) with *HERC2* rs12913832. These four SNPs achieved prediction accuracies for blue and brown eye color using a tree model approach in which the genotypes of each SNP drive the prediction towards one color category (blue, brown, intermediate) until a terminal node is reached [35].

In the same year, Ruiz et al. [36] described a test based on 23 SNPs, tested in Europeans, obtaining eye color phenotypes from a subset of 13 SNP genotypes via a Bayesian classifier (Snipper). Of these, six were the same IrisPlex SNPs, four were among *HERC2* SNPs (rs1129038, rs11636232, rs7183877, and rs1667394), and the other three were among *OCA2* SNPs (rs4778241, rs4778232, and rs8024968), showing AUC values of 0.999 for blue, 0.990 for brown, and 0.816 for intermediate eyes (comprising green and hazel) (Table 3).

Another predictive algorithm was developed using five SNPs (rs12913832, rs12203592, rs16891982, rs6119471, and rs12896399) in a sample of European, non-European, and mixed-ancestry populations [37] (Table 3). With this system, however, a positive color prediction cannot always be achieved, and wider “non-color” (not-blue and not-brown) outcomes are possible.

In view of a prospective application to forensic casework, only the IrisPlex has been developed and tested in several European and non-European populations, producing accurate predictions in different populations [74,75,78,79]. However, the method also showed limitations in predicting intermediate phenotypes, affecting the overall performance when intermediates are particularly frequent; for this reason, it was shown that the inclusion of *HERC2* rs1129038 improves intermediate (green-hazel) eye color prediction [80]. Further studies in eye color prediction are necessary to better understand the role of SNPs in LD, such as those from the *HERC2–OCA2* region.

In 2017, due to the great prediction power of FDP, the visible attributes through GEnomics (VISAGE) Consortium (https://www.visage-h2020.eu/ accessed on 2 June 2023) was founded. The main goal of the VISAGE Consortium was to develop and validate new and reliable molecular and statistical tools to predict appearance, ancestry, and age by using DNA-based technology [81]. The VISAGE Consortium validated also the “VISAGE Enhanced Tool for Appearance and Ancestry inference from DNA”, the first forensic-driven genetic laboratory tool that comprises well-established markers (about 524 SNPs) for eye, hair, and skin color with more recently discovered DNA markers for eyebrow color, freckling, hair shape, male pattern baldness, and bio-geographic ancestry-informative DNA markers [82,83]. The aim of the VISAGE Consortium is not only to evaluate new markers for forensic appearance prediction but also to supervise methodology applications and ethical implications for the huge amount of phenotypical and personal data that could be evaluated during the new FDP analysis [84,85].

An Italian study attempted to introduce a new approach with the aim of improving eye color prediction using the well-validated IrisPlex system, but more research is still needed to improve its prediction accuracy, especially for intermediate eye color [86]. To further improve the prediction of human eye color, quantitative characterization of eye color phenotypes has been adopted using high-resolution photographic images of the iris in combination with DIAT software analysis, with the aim of increasing understanding of human variability in this character. Advanced machine learning approaches, such as the progress on the implementation of MPS for forensic purposes, i.e., phenotypic trait prediction in ancient DNA analysis, showed a significant increase in sensitivity for color prediction [87,88,89,90]. Another challenge may be the development of universal genomic predictive methods with the goal of collecting sufficiently large data sets using whole-genome sequencing (WGS) technology and enabling the identification of rare DNA variants implicated in phenotype determination [91]. Recently, to identify new eye color variants by MPS to be added to the *HERC2* SNP rs12913832, currently the best-known predictor for blue and brown eye color, exceptions were found in individuals with rs12913832:AA/AG who did not have the expected brown eye color. New candidate variants were identified for explaining blue eye color in individuals with the rs12913832:AA and AG genotypes, suggesting inclusion of them in future prediction models [92].

## 5. Conclusions

Previous genome-wide association studies (GWAS) have identified several SNPs that were significantly associated with eye color [26,93], a polygenic trait that, in the past, was assumed to be genetically simple [94]. The strongest genetic influence on eye color is exerted by the *HERC2*-*OCA2* locus, where intronic SNPs in *HERC2* interact with the *OCA2* promoter via chromatin-loop remodeling [25,27]. Although previous genetic knowledge allows for accurate prediction of blue and brown eye color with the IrisPlex system, nonblue and nonbrown eye color cannot be genetically predicted, presumably because of the unknown function of the responsible genes and their predictive SNPs [29,74].

Intriguingly, DNA variants related to eye color are largely in non-coding regions of the involved genes, and it remains largely unknown if and how these non-coding DNA variants influence phenotypic traits at the molecular level. Due to limited knowledge on genes and predictive DNA markers, only some of these non-coding DNA variants constitute potential regulatory elements for distal genes, and more studies are needed to understand how other non-coding DNA markers can carry the same pigmentation information as coding ones, especially for intermediate colors, following an approach that also includes a quantitative measurement of the melanin content. Indeed, several DNA markers used in the above predictive systems are regulatory SNPs located in introns or intergenic regions, such as the most eye color predictive SNP, *HERC2* rs12913832. For forensic practices, this means that DNA-based iris color prediction needs to be combined with a DNA test that allows inferring the other pigmentation traits (fully or partially) of the unknown person with high accuracy. However, the current knowledge of all genetic factors involved in skin, eye, and hair color is far from complete.

To enhance our knowledge on color eye formation and its DNA-based prediction, it is necessary to understand the molecular basis of eye color formation related to the main polymorphisms involved in the determination of iris pigmentation [95,96], especially for the SNPs in non-coding regions that seem to be the most predictive for this phenotype. Among the 14 SNPs currently used in the main forensic DNA prediction systems (see Table 3), only two are missense mutations (in the *OCA2* and *SLC45A2* genes), with the large majority (11 SNPs) being in the introns and one (in the *HERC2* gene) corresponding to a synonymous substitution. The SNP rs12913832 in the *HERC2* gene is well known for its regulatory activity in the *OCA2* gene, but for the other SNPs, we lack precise information. One might think of studying these polymorphisms with different molecular approaches using the melanocytic cell lines currently available, which may constitute a good in vitro model to evaluate the genomic properties of the above SNPs in relation to the organization of the chromatin in the interphase cell nucleus and its influence on the eye color phenotype, or by genetic engineering methodologies (such as CRISPR/Cas9), which allow to introduce specific genetic modifications in the nucleotides of the SNPs relevant to the eye color and evaluate the phenotypic effects.

## Figures and Tables

**Table 1:** Genes and SNPs related to eye color.

Gene	SNPs (No.)	SNP Location				
		5′-UTR	3′-UTR	Intron	N.Syn.	Syn.
*ASIP*	3	1	1	1		
*HERC2*	10			9		1
*IRF4*	1			1		
*MC1R*	5				5	
*OCA2*	42			36	5	1
*SLC24A4*	1	1				
*SLC24A5*	1				1	
*SLC45A2*	2				2	
*TYR*	2			1	1	
*TYRP1*	6	3	1	1		1

N.Syn: non synonymous variant; Syn: synonymous variant.

## Data Availability

Not applicable.

## References

[1] Jobling M.A., Gill P. (2004). Encoded evidence: DNA in forensic analysis. Nat. Rev. Genet..

[2] Schneider P.M., Prainsack B., Kayser M. (2019). The Use of Forensic DNA Phenotyping in Predicting Appearance and Biogeographic Ancestry. Dtsch. Arztebl. Int..

[3] Wienroth M. (2018). Governing anticipatory technology practices. Forensic DNA phenotyping and the forensic genetics community in Europe. New Genet. Soc..

[4] Oosthuizen T., Howes L.M. (2022). The development of forensic DNA analysis: New debates on the issue of fundamental human rights. Forensic Sci. Int. Genet..

[5] Hopman R., M’charek A. (2020). Facing the unknown suspect: Forensic DNA phenotyping and the oscillation between the individual and the collective. Biosocieties.

[6] M’charek A., Toom V., Jong L. (2020). The Trouble with Race in Forensic Identification. Sci. Technol. Hum. Values.

[7] Kayser M., Branicki W., Parson W., Phillips C. (2023). Recent advances in Forensic DNA Phenotyping of appearance, ancestry and age. Forensic Sci. Int. Genet..

[8] Ballard D., Winkler-Galicki J., Wesoły J. (2020). Massive parallel sequencing in forensics: Advantages, issues, technicalities, and prospects. Int. J. Leg. Med..

[9] Butler J.M. (2022). Recent advances in forensic biology and forensic DNA typing: INTERPOL review 2019–2022. Forensic Sci. Int. Synerg..

[10] Carratto T.M.T., Moraes V.M.S., Recalde T.S.F., de Oliveira M.L.G., Mendes-Junior C.T. (2022). Applications of massively parallel sequencing in forensic genetics. Genet. Mol. Biol..

[11] Fabbri M., Alfieri L., Mazdai L., Frisoni P., Gaudio R.M., Neri M. (2023). Application of Forensic DNA Phenotyping for Prediction of Eye, Hair and Skin Colour in Highly Decomposed Bodies. Healthcare.

[12] Haddrill P.R. (2021). Developments in forensic DNA analysis. Emerg. Top. Life Sci..

[13] Pajnič I.Z., Zupanc T., Leskovar T., Črešnar M., Fattorini P. (2022). Eye and Hair Color Prediction of Ancient and Second World War Skeletal Remains Using a Forensic PCR-MPS Approach. Genes.

[14] Melchionda F., Silvestrini B., Robino C., Bini C., Fattorini P., Martinez-Labarga C., De Angelis F., Tagliabracci A., Turchi C. (2022). Development and Validation of MPS-Based System for Human Appearance Prediction in Challenging Forensic Samples. Genes.

[15] Diepenbroek M., Bayer B., Anslinger K. (2021). Pushing the Boundaries: Forensic DNA Phenotyping Challenged by Single-Cell Sequencing. Genes.

[16] Montesanto A., D’aquila P., Lagani V., Paparazzo E., Geracitano S., Formentini L., Giacconi R., Cardelli M., Provinciali M., Bellizzi D. (2020). A New Robust Epigenetic Model for Forensic Age Prediction. J. Forensic Sci..

[17] Onofri M., Delicati A., Marcante B., Carlini L., Alessandrini F., Tozzo P., Carnevali E. (2023). Forensic Age Estimation through a DNA Methylation-Based Age Prediction Model in the Italian Population: A Pilot Study. Int. J. Mol. Sci..

[18] Alshehhi A., Almarzooqi A., Alhammadi K., Werghi N., Tay G.K., Alsafar H. (2023). Advancement in Human Face Prediction Using DNA. Genes.

[19] Kaiser M. (2015). Forensic DNA Phenotyping: Predicting human appearance from crime scene material for investigative purposes. Forensic Sci. Int. Genet..

[20] Maroñas O., Söchtig J., Ruiz Y., Phillips C., Carracedo A., Lareu M.V. (2015). The genetics of skin, hair, and eye color variation and its relevance to forensic pigmentation predictive tests. Forensic Sci. Rev..

[21] Dorgaleleh S., Naghipoor K., Barahouie A., Dastaviz F., Oladnabi M. (2020). Molecular and biochemical mechanisms of human iris color: A comprehensive review. J. Cell. Physiol..

[22] D’Mello S.A.N., Finlay G.J., Baguley B.C., Askarian-Amiri M.E. (2016). Signaling Pathways in Melanogenesis. Int. J. Mol. Sci..

[23] Duffy D.L., Montgomery G.W., Chen W., Zhao Z.Z., Le L., James M.R., Hayward N.K., Martin N.G., Sturm R.A. (2007). A three-single-nucleotide polymorphism haplotype in intron 1 of OCA2 explains most human eye-color variation. Am. J. Hum. Genet.

[24] Kayser M., Liu F., Janssens A.C.J., Rivadeneira F., Lao O., van Duijn K., Vermeulen M., Arp P., Jhamai M.M., van IJcken W.F. (2008). Three genome-wide association studies and a linkage analysis identify HERC2 as a human iris color gene. Am. J. Hum. Genet..

[25] Sturm R.A., Duffy D.L., Zhao Z.Z., Leite F.P., Stark M.S., Hayward N.K., Martin N.G., Montgomery G.W. (2008). A Single SNP in an Evolutionary Conserved Region within Intron 86 of the HERC2 Gene Determines Human Blue-Brown Eye Color. Am. J. Hum. Genet..

[26] Sulem P., Gudbjartsson D.F., Stacey S.N., Helgason A., Rafnar T., Magnusson K.P., Manolescu A., Karason A., Palsson A., Thorleifsson G. (2007). Genetic determinants of hair, eye and skin pigmentation in Europeans. Nat. Genet..

[27] Visser M., Kayser M., Palstra R.-J. (2012). HERC2 rs12913832 modulates human pigmentation by attenuating chromatin-loop formation between a long-range enhancer and the OCA2 promoter. Genome Res..

[28] Frudakis T., Terravainen T., Thomas M. (2007). Multilocus OCA2 genotypes specify human iris colors. Hum. Genet..

[29] Liu F., van Duijn K., Vingerling J.R., Hofman A., Uitterlinden A.G., Janssens A.C., Kayser M. (2009). Eye color and the prediction of complex phenotypes from genotypes. Curr. Biol..

[30] Lee S.-T., Nicholls R.D., Jong M.T., Fukai K., Spritz R.A. (1995). Organization and sequence of the human P gene and identification of a new family of transport proteins. Genomics.

[31] Toyofuku K., Valencia J.C., Kushimoto T., Costin G.-E., Virador V.M., Vieira W.D., Ferrans V.J., Hearing V.J. (2002). The Etiology of Oculocutaneous Albinism (OCA) Type II: The Pink Protein Modulates the Processing and Transport of Tyrosinase. Pigment. Cell Res..

[32] Sturm R.A., Larsson M. (2009). Genetics of human iris colour and patterns. Pigment. Cell Melanoma Res..

[33] Sturm R.A., Frudakis T.N. (2004). Eye colour: Portals into pigmentation genes and ancestry. Trends Genet..

[34] Walsh S., Lindenbergh A., Zuniga S.B., Sijen T., de Knijff P., Kayser M., Ballantyne K.N. (2011). Developmental validation of the IrisPlex system: Determination of blue and brown iris colour for forensic intelligence. Forensic Sci. Int. Genet..

[35] Allwood J.S., Harbison S. (2013). SNP model development for the prediction of eye colour in New Zealand. Forensic Sci. Int. Genet..

[36] Ruiz Y., Phillips C., Gomez-Tato A., Alvarez-Dios J., de Cal M.C., Cruz R., Maroñas O., Söchtig J., Fondevila M., Rodriguez-Cid M. (2013). Further development of forensic eye color predictive tests. Forensic Sci. Int. Genet..

[37] Hart K.L., Kimura S.L., Mushailov V., Budimlija Z.M., Prinz M., Wurmbach E. (2013). Improved eye- and skin-color prediction based on 8 SNPs. Croat. Med. J..

[38] Andersen J.D., Johansen P., Harder S., Christoffersen S.R., Delgado M.C., Henriksen S.T., Nielsen M.M., Sørensen E., Ullum H., Hansen T. (2013). Genetic analyses of the human eye colours using a novel objective method for eye colour classification. Forensic Sci. Int. Genet..

[39] Pośpiech E., Wojas-Pelc A., Walsh S., Liu F., Maeda H., Ishikawa T., Skowron M., Kayser M., Branicki W. (2014). The common occurrence of epistasis in the determination of human pigmentation and its impact on DNA-based pigmentation phenotype prediction. Forensic Sci. Int. Genet..

[40] Galligan J.T., Martinez-Noël G., Arndt V., Hayes S., Chittenden T.W., Harper J.W., Howley P.M. (2015). Proteomic Analysis and Identification of Cellular Interactors of the Giant Ubiquitin Ligase HERC2. J. Proteome Res..

[41] Eiberg H., Troelsen J., Nielsen M., Mikkelsen A., Mengel-From J., Kjaer K.W., Hansen L. (2008). Blue eye color in humans may be caused by a perfectly associated founder mutation in a regulatory element located within the HERC2 gene inhibiting OCA2 expression. Hum. Genet..

[42] Giebel L.B., Strunk K.M., Spritz R.A. (1991). Organization and nucleotide sequences of the human tyrosinase gene and a truncated tyrosinase-related segment. Genomics.

[43] Wang N., Hebert D.N. (2006). Tyrosinase maturation through the mammalian secretory pathway: Bringing color to life. Pigment. Cell Res..

[44] Candille S.I., Absher D.M., Beleza S., Bauchet M., McEvoy B., Garrison N.A., Li J.Z., Myers R.M., Barsh G.S., Tang H. (2012). Genome-Wide Association Studies of Quantitatively Measured Skin, Hair, and Eye Pigmentation in Four European Populations. PLoS ONE.

[45] Alaluf S., Barrett K., Blount M., Carter N. (2003). Ethnic Variation in Tyrosinase and TYRP1 Expression in Photoexposed and Photoprotected Human Skin. Pigment. Cell Res..

[46] Meyer O.S., Lunn M.M.B., Garcia S.L., Kjærbye A.B., Morling N., Børsting C., Andersen J.D. (2020). Association between brown eye colour in rs12913832:GG individuals and SNPs in TYR, TYRP1, and SLC24A4. PLoS ONE.

[47] Harada M., Li Y.F., El-Gamil M., Rosenberg S.A., Robbins P.F. (2001). Use of an in vitro immunoselected tumor line to identify shared melanoma antigens recognized by HLA-A*0201-restricted T cells. Cancer Res.

[48] Graf J., Hodgson R., van Daal A. (2005). Single nucleotide polymorphisms in theMATP gene are associated with normal human pigmentation variation. Hum. Mutat..

[49] Newton J., Cohen-Barak O., Hagiwara N., Gardner J.M., Davisson M.T., King R.A., Brilliant M.H. (2001). Mutations in the Human Orthologue of the Mouse underwhite Gene (uw) Underlie a New Form of Oculocutaneous Albinism, OCA4. Am. J. Hum. Genet..

[50] Du J., Fisher D.E. (2002). Identification of Aim-1 as the underwhiteMouse Mutant and Its Transcriptional Regulation by MITF. J. Biol. Chem..

[51] Nakayama K., Fukamachi S., Kimura H., Koda Y., Soemantri A., Ishida T. (2002). Distinctive distribution of AIM1 polymorphism among major human populations with different skin color. J. Hum. Genet..

[52] Fernandez L., Milne R., Pita G., Avilés J., Lázaro P., Benítez J., Ribas G. (2008). *SLC45A2*: A novel malignant melanoma-associated gene. Hum. Mutat..

[53] Pośpiech E., Draus-Barini J., Kupiec T., Wojas-Pelc A., Branicki W. (2011). Gene–gene interactions contribute to eye colour variation in humans. J. Hum. Genet..

[54] Lamason R.L., Mohideen M.A.P., Mest J.R., Wong A.C., Norton H.L., Aros M.C., Jurynec M.J., Mao X., Humphreville V.R., Humbert J.E. (2005). SLC24A5, a putative cation exchanger, affects pigmentation in zebrafish and humans. Science.

[55] Soejima M., Koda Y. (2007). Population differences of two coding SNPs in pigmentation-related genes SLC24A5 and SLC45A2. Int. J. Leg. Med..

[56] Cook A.L., Chen W., Thurber A.E., Smit D.J., Smith A.G., Bladen T.G., Brown D.L., Duffy D.L., Pastorino L., Bianchi-Scarra G. (2009). Analysis of cultured human melanocytes based on polymorphisms within the SLC45A2/MATP, SLC24A5/NCKX5, and OCA2/P loci. J. Investig. Dermatol..

[57] Canfield V.A., Berg A., Peckins S., Wentzel S.M., Ang K.C., Oppenheimer S., Cheng K.C. (2013). Molecular Phylogeography of a Human Autosomal Skin Color Locus Under Natural Selection. G3.

[58] Wilson B.D., Ollmann M.M., Kang L., Stoffel M., Bell G.I., Barsh G.S. (1995). Structure and function of ASP, the human homolog of the mouse agouti gene. Hum. Mol. Genet..

[59] Voisey J., Kelly G., Van Daal A. (2003). Agouti Signal Protein Regulation in Human Melanoma Cells. Pigment. Cell Res..

[60] Zeigler-Johnson C., Panossian S., Gueye S.M., Jalloh M., Ofori-Adjei D., Kanetsky P.A. (2004). Population Differences in the Frequency of the Agouti Signaling Protein g.8818A>G Polymorphism. Pigment. Cell Res..

[61] Kanetsky P.A., Swoyer J., Panossian S., Holmes R., Guerry D., Rebbeck T.R. (2002). A Polymorphism in the Agouti Signaling Protein Gene Is Associated with Human Pigmentation. Am. J. Hum. Genet..

[62] Harding R.M., Healy E., Ray A.J., Ellis N.S., Flanagan N., Todd C., Dixon C., Sajantila A., Jackson I.J., Birch-Machin M.A. (2000). Evidence for variable selective pressures at MC1R. Am. J. Hum. Genet..

[63] Palmer J.S., Duffy D.L., Box N.F., Aitken J.F., O’Gorman L.E., Green A.C., Hayward N.K., Martin N.G., Sturm R.A. (2000). Melanocortin-1 receptor polymorphisms and risk of melanoma: Is the association explained solely by pigmentation phenotype?. Am. J. Hum. Genet..

[64] Valverde P., Healy E., Jackson I., Rees J.L., Thody A.J. (1995). Variants of the melanocyte–stimulating hormone receptor gene are associated with red hair and fair skin in humans. Nat. Genet..

[65] Frändberg P.-A., Doufexis M., Kapas S., Chhajlani V. (1998). Human Pigmentation Phenotype: A Point Mutation Generates Nonfunctional MSH Receptor. Biochem. Biophys. Res. Commun..

[66] Cone R.D., Lu D., Koppula S., Våge D.I., Klungland H., Boston B., Chen W., Orth D.N., Pouton C., Kesterson R.A. (1996). The melanocortin receptors: Agonists, antagonists, and the hormonal control of pigmentation. Recent Prog. Horm. Res..

[67] Makova K., Norton H. (2005). Worldwide polymorphism at the MC1R locus and normal pigmentation variation in humans. Peptides.

[68] Paun A., Pitha P.M. (2007). The IRF family, revisited. Biochimie.

[69] Han J., Kraft P., Nan H., Guo Q., Chen C., Qureshi A., Hankinson S.E., Hu F.B., Duffy D.L., Zhao Z.Z. (2008). A Genome-Wide Association Study Identifies Novel Alleles Associated with Hair Color and Skin Pigmentation. PLoS Genet..

[70] Praetorius C., Grill C., Stacey S.N., Metcalf A.M., Gorkin D.U., Robinson K.C., Van Otterloo E., Kim R.S., Bergsteinsdottir K., Ogmundsdottir M.H. (2013). A Polymorphism in IRF4 Affects Human Pigmentation through a Tyrosinase-Dependent MITF/TFAP2A Pathway. Cell.

[71] Visser M., Palstra R.-J., Kayser M. (2015). Allele-specific transcriptional regulation of IRF4 in melanocytes is mediated by chromatin looping of the intronic rs12203592 enhancer to the IRF4 promoter. Hum. Mol. Genet..

[72] Valenzuela R.K., Henderson M.S., Walsh M.H., Garrison N.A., Kelch J.T., Cohen-Barak O., Erickson D.T., Meaney F.J., Walsh J.B., Cheng K.C. (2010). Predicting Phenotype from Genotype: Normal Pigmentation. J. Forensic Sci..

[73] Mengel-From J., Børsting C., Sanchez J.J., Eiberg H., Morling N. (2010). Human eye colour and HERC2, OCA2 and MATP. Forensic Sci. Int. Genet..

[74] Walsh S., Liu F., Ballantyne K.N., van Oven M., Lao O., Kayser M. (2011). IrisPlex: A sensitive DNA tool for accurate prediction of blue and brown eye colour in the absence of ancestry information. Forensic Sci. Int. Genet..

[75] Walsh S., Wollstein A., Liu F., Chakravarthy U., Rahu M., Seland J.H., Soubrane G., Tomazzoli L., Topouzis F., Vingerling J.R. (2012). DNA-based eye colour prediction across Europe with the IrisPlex system. Forensic Sci. Int. Genet..

[76] Chaitanya L., Walsh S., Andersen J.D., Ansell R., Ballantyne K., Ballard D., Banemann R., Bauer C.M., Bento A.M., Brisighelli F. (2014). Collaborative EDNAP exercise on the IrisPlex system for DNA-based prediction of human eye colour. Forensic Sci. Int. Genet..

[77] Keating B., Bansal A.T., Walsh S., Millman J., Newman J., Kidd K., Budowle B., Eisenberg A., Donfack J. (2013). First all-in-one diagnostic tool for DNA intelligence: Genome-wide inference of biogeographic ancestry, appearance, relatedness, and sex with the Identitas v1 Forensic Chip. Int. J. Leg. Med..

[78] Walsh S., Chaitanya L., Clarisse L., Wirken L., Draus-Barini J., Kovatsi L., Maeda H., Ishikawa T., Sijen T., de Knijff P. (2014). Developmental validation of the HIrisPlex system: DNA-based eye and hair colour prediction for forensic and anthropological usage. Forensic Sci. Int. Genet..

[79] Sari O.I., Simsek S.Z., Filoglu G., Bulbul O. (2022). Predicting Eye and Hair Color in a Turkish Population Using the HIrisPlex System. Genes.

[80] Freire-Aradas A., Ruiz Y., Phillips C., Maroñas O., Söchtig J., Tato A.G., Dios J., de Cal M.C., Silbiger V., Luchessi A. (2014). Exploring iris colour prediction and ancestry inference in admixed populations of South America. Forensic Sci. Int. Genet..

[81] Xavier C., de la Puente M., Mosquera-Miguel A., Freire-Aradas A., Kalamara V., Ralf A., Revoir A., Gross T., Schneider P., Ames C. (2022). Development and inter-laboratory evaluation of the VISAGE Enhanced Tool for Appearance and Ancestry inference from DNA. Forensic Sci. Int. Genet..

[82] Ruiz-Ramírez J., de la Puente M., Xavier C., Ambroa-Conde A., Álvarez-Dios J., Freire-Aradas A., Mosquera-Miguel A., Ralf A., Amory C., Katsara M. (2023). Development and evaluations of the ancestry informative markers of the VISAGE Enhanced Tool for Appearance and Ancestry. Forensic Sci. Int. Genet..

[83] Palencia-Madrid L., Xavier C., De La Puente M., Hohoff C., Phillips C., Kayser M., Parson W. (2020). Evaluation of the VISAGE Basic Tool for Appearance and Ancestry Prediction Using PowerSeq Chemistry on the MiSeq FGx System. Genes.

[84] Xavier C., de la Puente M., Mosquera-Miguel A., Freire-Aradas A., Kalamara V., Vidaki A., Gross T.E., Revoir A., Pośpiech E., Kartasińska E. (2020). Development and validation of the VISAGE AmpliSeq basic tool to predict appearance and ancestry from DNA. Forensic Sci. Int. Genet..

[85] Zieger M. (2022). Forensic DNA phenotyping in Europe: How far may it go?. J. Law. Biosci..

[86] Paparazzo E., Gozalishvili A., Lagani V., Geracitano S., Bauleo A., Falcone E., Passarino G., Montesanto A. (2022). A new approach to broaden the range of eye colour identifiable by IrisPlex in DNA phenotyping. Sci. Rep..

[87] Gross T.E., Fleckhaus J., Schneider P.M. (2021). Progress in the implementation of massively parallel sequencing for forensic genetics: Results of a European-wide survey among professional users. Int. J. Leg. Med..

[88] Kukla-Bartoszek M., Teisseyre P., Pośpiech E., Karłowska-Pik J., Zieliński P., Woźniak A., Boroń M., Dąbrowski M., Zubańska M., Jarosz A. (2021). Searching for improvements in predicting human eye colour from DNA. Int. J. Leg. Med..

[89] Ragazzo M., Puleri G., Errichiello V., Manzo L., Luzzi L., Potenza S., Strafella C., Peconi C., Nicastro F., Caputo V. (2021). Evaluation of OpenArray™ as A Genotyping Method for Forensic DNA Phenotyping and Human Identification. Genes.

[90] Pajnič I.Z., Leskovar T., Črešnar M. (2023). Eye and hair color prediction of an early medieval adult and subadult skeleton using massive parallel sequencing technology. Int. J. Leg. Med..

[91] Pośpiech E., Teisseyre P., Mielniczuk J., Branicki W. (2022). Predicting Physical Appearance from DNA Data—Towards Genomic Solutions. Genes.

[92] Salvo N.M., Andersen J.D., Janssen K., Meyer O.L., Berg T., Børsting C., Olsen G.-H. (2023). Association between Variants in the *OCA2-HERC2* Region and Blue Eye Colour in *HERC2* rs12913832 AA and AG Individuals. Genes.

[93] Liu F., Visser M., Duffy D.L., Hysi P.G., Jacobs L.C., Lao O., Zhong K., Walsh S., Chaitanya L., Wollstein A. (2015). Genetics of skin color variation in Europeans: Genome-wide association studies with functional follow-up. Hum. Genet..

[94] Davenport G.C., Davenport C.B. (1907). Heredity of eye-color in man. Science.

[95] d’Ischia M., Wakamatsu K., Cicoira F., Di Mauro E., Garcia-Borron J.C., Commo S., Galván I., Ghanem G., Kenzo K., Meredith P. (2015). Melanins and melanogenesis: From pigment cells to human health and technological applications. Pigment. Cell Melanoma Res..

[96] Wakamatsu K., Ito S. (2023). Recent Advances in Characterization of Melanin Pigments in Biological Samples. Int. J. Mol. Sci..

